# Supplementary figures and images for: Individual reflectance of solar radiation confers a thermoregulatory benefit to dimorphic males bees (Centris pallida) using distinct microclimates
Source: PLoS One. 2023 Mar 14;18(3):e0271250. doi: 10.1371/journal.pone.0271250 (PMC10013911; doi:10.1371/journal.pone.0271250)

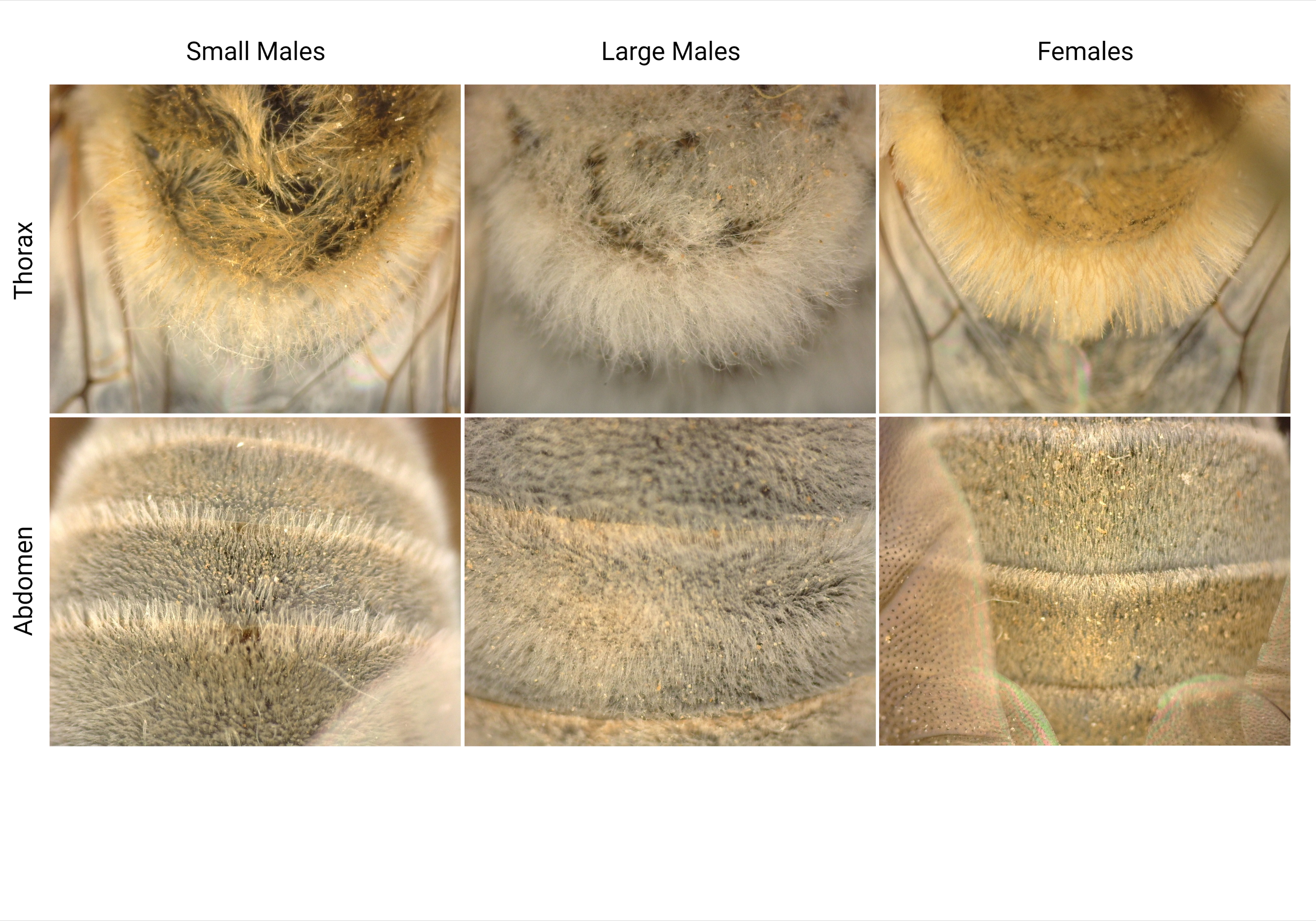

Supplement: S1 Fig — All photos taken at 85X magnification on a DinoLite AM4915ZT. (TIF) [file pone.0271250.s001.tif]

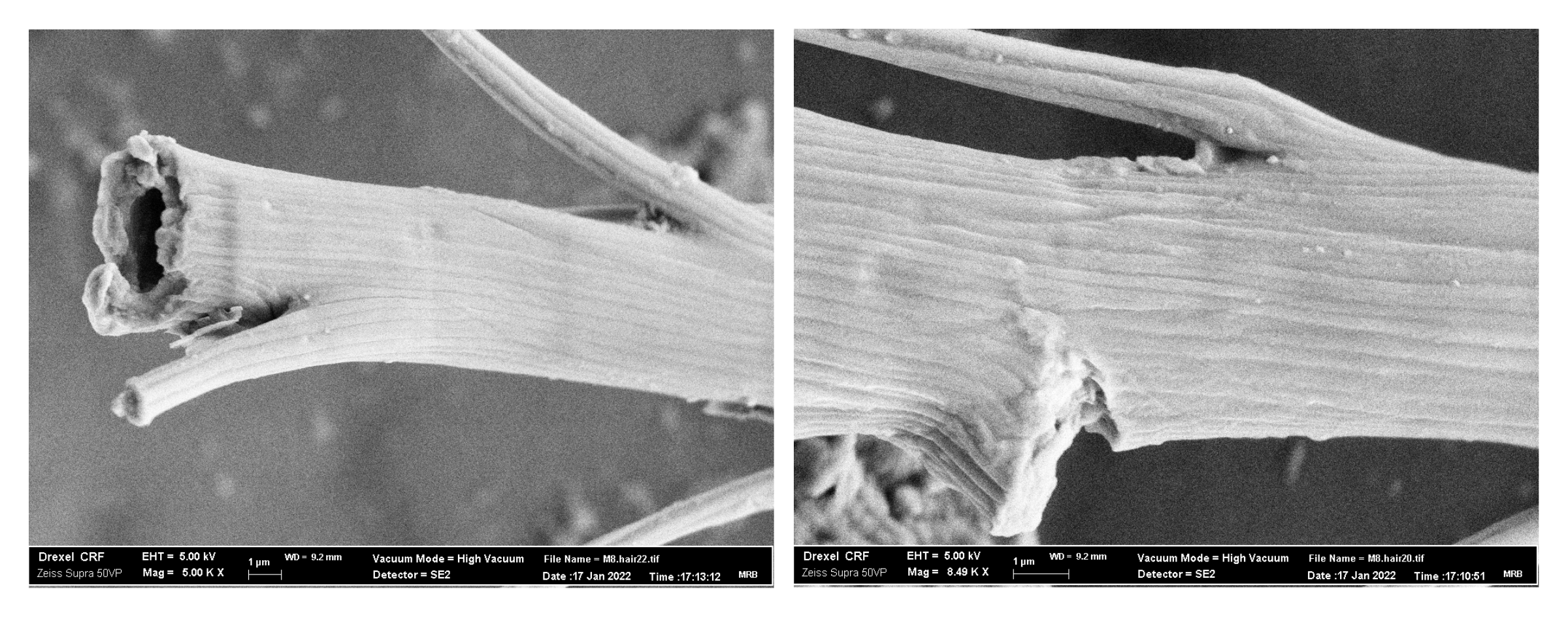

Supplement: S2 Fig — Left) The hairs of large-morph males have a round cross-section, and are covered in striations (5000X). Right) Close-up of striations on the exterior surface of large-morph male thorax hairs (8500X). The somewhat regular striations are reminiscent of the structure of the highly reflective hairs of the Saharan silver ant (in Shi et al. 2015). (TIF) [file pone.0271250.s002.tif]
