# Supplementary material for: Individual reflectance of solar radiation confers a thermoregulatory benefit to dimorphic males bees (Centris pallida) using distinct microclimates
Source: PLoS One. 2023 Mar 14;18(3):e0271250. doi: 10.1371/journal.pone.0271250 (PMC10013911; doi:10.1371/journal.pone.0271250)
Supplement: S1 Table — Mean ± SD of mean percent reflectance of small males, large males, and females in the UV (290–399), VIS (400–700), close NIR (701–1400) and far NIR (1401–2500) on dorsal surface of the abdomen and thorax (unshaved and shaved). (DOCX) [file pone.0271250.s003.docx]

**S1 Table. Mean percent reflectance of males and females in the UV-NIR.** Mean ± SD of mean percent reflectance of small males, large males, and females in the UV (290-399), VIS (400-700), close NIR (701-1400) and far NIR (1401-2500) on dorsal surface of the abdomen and thorax (unshaved and shaved).

| Morph | Wave-  lengths | Region | mean (%) | SD |
| --- | --- | --- | --- | --- |
| *Small male* | *UV*  VIS  *close NIR*  far NIR | *Abdomen (dorsal)*  *Thorax (dorsal, unshaved)*  *Thorax (dorsal, shaved)*  *Abdomen (ventral)*  Abdomen (dorsal)  Thorax (dorsal, unshaved)  Thorax (dorsal, shaved)  Abdomen (ventral)  *Abdomen (dorsal)*  *Thorax (dorsal, unshaved)*  *Thorax (dorsal, shaved)*  *Abdomen (ventral)*  Abdomen (dorsal)  Thorax (dorsal, unshaved)  Thorax (dorsal, shaved)  Abdomen (ventral) | *6.23*  *3.46*  *5.59*  *5.42*  12.15  11.61  5.85  9.68  *21.65*  *23.70*  *17.63*  *24.91*  27.41  29.80  25.14  30.53 | *1.00*  *1.26*  *0.51*  *0.84*  2.09  2.06  0.31  1.48  *3.97*  *2.82*  *0.99*  *3.78*  6.07  2.97  1.34  4.56 |
| *Large male* | *UV*  VIS  *close NIR*  far NIR | *Abdomen (dorsal)*  *Thorax (dorsal, unshaved)*  *Thorax (dorsal, shaved)*  *Abdomen (ventral)*  Abdomen (dorsal)  Thorax (dorsal, unshaved)  Thorax (dorsal, shaved)  Abdomen (ventral)  *Abdomen (dorsal)*  *Thorax (dorsal, unshaved)*  *Thorax (dorsal, shaved)*  *Abdomen (ventral)*  Abdomen (dorsal)  Thorax (dorsal, unshaved)  Thorax (dorsal, shaved)  Abdomen (ventral) | *10.45*  *10.08*  *5.66*  *5.00*  19.26  19.03  5.84  11.11  *33.77*  *29.51*  *16.00*  *27.01*  43.34  35.59  22.10  32.99 | *2.68*  *2.29*  *1.14*  *1.49*  4.05  2.32  0.48  2.69  *5.19*  *2.69*  *2.10*  *5.36*  7.17  3.09  2.76  7.37 |
| *Female* | *UV*  VIS  *close NIR*  far NIR | *Abdomen (dorsal)*  *Thorax (dorsal, unshaved)*  *Thorax (dorsal, shaved)*  *Abdomen (ventral)*  Abdomen (dorsal)  Thorax (dorsal, unshaved)  Thorax (dorsal, shaved)  Abdomen (ventral)  *Abdomen (dorsal)*  *Thorax (dorsal, unshaved)*  *Thorax (dorsal, shaved)*  *Abdomen (ventral)*  Abdomen (dorsal)  Thorax (dorsal, unshaved)  Thorax (dorsal, shaved)  Abdomen (ventral) | *7.68*  *5.69*  *6.41*  *3.59*  17.82  14.46  6.37  6.35  *33.52*  *26.33*  *16.94*  *27.28*  42.09  32.36  24.42  34.33 | *0.93*  *1.06*  *0.38*  *1.03*  1.47  2.47  0.49  0.93  *2.47*  *3.25*  *1.32*  *3.51*  3.82  3.47  2.17  4.93 |
