# Supplementary material for: Individual reflectance of solar radiation confers a thermoregulatory benefit to dimorphic males bees (Centris pallida) using distinct microclimates
Source: PLoS One. 2023 Mar 14;18(3):e0271250. doi: 10.1371/journal.pone.0271250 (PMC10013911; doi:10.1371/journal.pone.0271250)
Supplement: S2 Table — (DOCX) [file pone.0271250.s004.docx]

**S2 Table.** Tukey’s multiple comparisons test (following two-way RM ANOVA with Geisser-Greenhouse Correction) of mean reflectance of small males (SM), large males (LM), and females (F) in the UV (290-399), VIS (400-700), close NIR (701-1400) and far NIR (1401-2500) on dorsal surface of the abdomen and thorax (unshaved and shaved).

| Comparison Region | Wave-  lengths | Comparison Groups | q | df | adjusted p value |
| --- | --- | --- | --- | --- | --- |
| *Abdomen - Dorsal* | *UV* |  |  |  |  |
|  |  | **LM vs. SM** | 6.60 | 11.47 | **0.0182** |
|  |  | LM vs. F | 4.35 | 11.14 | 0.20 |
|  |  | SM vs. F | 4.77 | 17.90 | 0.10 |
|  | *VIS* |  |  |  |  |
|  |  | **LM vs. SM** | 6.99 | 13.48 | **0.0084** |
|  |  | LM vs. F | 1.50 | 11.34 | > 0.99 |
|  |  | **SM vs. F** | 9.92 | 16.17 | **0.0001** |
|  | *close NIR* |  |  |  |  |
|  |  | **LM vs. SM** | 8.29 | 16.84 | **0.0008** |
|  |  | LM vs. F | 0.20 | 12.88 | > 0.99 |
|  |  | **SM vs. F** | 11.35 | 15.07 | **< 0.0001** |
|  | *far NIR* |  |  |  |  |
|  |  | **LM vs. SM** | 7.58 | 17.53 | **0.0020** |
|  |  | LM vs. F | 0.69 | 13.73 | > 0.99 |
|  |  | **SM vs. F** | 9.15 | 15.16 | **0.0004** |
| *Thorax w/Hair* | *UV* |  |  |  |  |
|  |  | **LM vs. SM** | 11.32 | 14 | **< 0.0001** |
|  |  | **LM vs. F** | 7.78 | 12.69 | **0.0039** |
|  |  | **SM vs. F** | 6.05 | 17.49 | **0.0174** |
|  | *VIS* |  |  |  |  |
|  |  | **LM vs. SM** | 10.69 | 17.74 | **< 0.0001** |
|  |  | **LM vs. F** | 6.03 | 17.93 | **0.0173** |
|  |  | SM vs. F | 3.96 | 17.42 | 0.26 |
|  | *close NIR* |  |  |  |  |
|  |  | **LM vs. SM** | 6.67 | 17.97 | **0.0069** |
|  |  | LM vs. F | 3.37 | 17.41 | 0.46 |
|  |  | SM vs. F | 2.74 | 17.64 | 0.73 |
|  | *far NIR* |  |  |  |  |
|  |  | **LM vs. SM** | 6.04 | 17.97 | **0.0170** |
|  |  | LM vs. F | 3.11 | 17.77 | 0.57 |
|  |  | SM vs. F | 2.50 | 17.58 | 0.81 |
| *Thorax Shaved* | *UV* |  |  |  |  |
|  |  | LM vs. SM | 0.24 | 12.5 | > 0.99 |
|  |  | LM vs. F | 2.78 | 11.01 | 0.71 |
|  |  | **SM vs. F** | 5.70 | 16.66 | **0.0301** |
|  | *VIS* |  |  |  |  |
|  |  | LM vs. SM | 0.05 | 15.30 | > 0.99 |
|  |  | LM vs. F | 3.42 | 17.98 | 0.44 |
|  |  | SM vs. F | 3.99 | 14.99 | 0.26 |
|  | *close NIR* |  |  |  |  |
|  |  | LM vs. SM | 3.14 | 12.85 | 0.56 |
|  |  | LM vs. F | 1.70 | 15.17 | 0.98 |
|  |  | SM vs. F | 1.87 | 16.72 | 0.96 |
|  | *far NIR* |  |  |  |  |
|  |  | LM vs. SM | 4.44 | 13.00 | 0.17 |
|  |  | LM vs. F | 2.96 | 17.05 | 0.63 |
|  |  | SM vs. F | 1.26 | 14.97 | > 0.99 |
|  |  |  |  |  |  |
