# Supplementary material for: Individual reflectance of solar radiation confers a thermoregulatory benefit to dimorphic males bees (Centris pallida) using distinct microclimates
Source: PLoS One. 2023 Mar 14;18(3):e0271250. doi: 10.1371/journal.pone.0271250 (PMC10013911; doi:10.1371/journal.pone.0271250)
Supplement: S3 Table — (DOCX) [file pone.0271250.s005.docx]

**S3 Table.** Sidak’s multiple comparisons test (following two-way RM ANOVA with Geisser-Greenhouse Correction) of mean reflectance of the thorax, shaved versus unshaved, and abdomen, dorsal versus ventral, at different wavelengths (UV [290-399], VIS [400-700], close NIR [701-1400] and far NIR [1401-2500]) in large males or small males.

| Morph | Comparison | Wave  length | t | df | adjusted p value |
| --- | --- | --- | --- | --- | --- |
| Large Males - | Shaved vs. Unshaved |  |  |  |  |
| Thorax |  | UV | 5.46 | 13.22 | 0.0004 |
|  |  | VIS | 17.59 | 9.76 | < 0.0001 |
|  |  | cNIR | 12.52 | 16.97 | < 0.0001 |
|  |  | fNIR | 10.30 | 17.77 | < 0.0001 |
| Small Males - | Shaved vs. Unshaved |  |  |  |  |
| Thorax |  | UV | 4.95 | 11.91 | 0.0014 |
|  |  | VIS | 8.77 | 9.40 | < 0.0001 |
|  |  | cNIR | 6.43 | 11.21 | 0.0002 |
|  |  | fNIR | 4.53 | 12.50 | 0.0025 |
| Large Males - | Dorsal vs. Ventral |  |  |  |  |
| Abdomen |  | UV | 5.61 | 14.06 | 0.0003 |
|  |  | VIS | 5.31 | 15.66 | 0.0003 |
|  |  | cNIR | 2.82 | 17.93 | 0.0450 |
|  |  | fNIR | 3.19 | 17.99 | 0.0204 |
